# Mutant p53 in cancer: from molecular mechanism to therapeutic modulation

**DOI:** 10.1038/s41419-022-05408-1

**Published:** 2022-11-18

**Authors:** Xiaohua Chen, Taotao Zhang, Wei Su, Zhihui Dou, Dapeng Zhao, Xiaodong Jin, Huiwen Lei, Jing Wang, Xiaodong Xie, Bo Cheng, Qiang Li, Hong Zhang, Cuixia Di

**Affiliations:** 1 Bio-Medical Research Center, Institute of Modern Physics, Chinese Academy of Sciences, Lanzhou, 730000 China; 2 Advanced Energy Science and Technology Guangdong Laboratory, Huizhou, 516029 China; 3 Key Laboratory of Heavy Ion Radiation Biology and Medicine of Chinese Academy of Sciences, Lanzhou, 730000 China; 4 College of Life Sciences, University of Chinese Academy of Sciences, Beijing, 101408 China; 5 School of Nuclear Science and Technology, University of Chinese Academy of Sciences, Beijing, 101408 China; 6 School of Basic Medical Sciences, Lanzhou University, Lanzhou, 730000 China; 7 School of Life Sciences, Lanzhou University, Lanzhou, 730000 China; 8 Lanhai Neclear Medical Research Center, Putian, 351100 China

**Keywords:** Oncogenes, Cancer

## Abstract

*TP53*, a crucial tumor suppressor gene, is the most commonly mutated gene in human cancers. Aside from losing its tumor suppressor function, mutant p53 (mutp53) often acquires inherent, novel oncogenic functions, which is termed “gain-of-function”. Emerging evidence suggests that mutp53 is highly associated with advanced malignancies and poor prognosis, which makes it a target for development of novel cancer therapies. Herein, we provide a summary of our knowledge of the mutp53 types and mutp53 spectrum in cancers. The mechanisms of mutp53 accumulation and gain-of-function are also summarized. Furthermore, we discuss the gain-of-function of mutp53 in cancers: genetic instability, ferroptosis, microenvironment, and stemness. Importantly, the role of mutp53 in the clinic is also discussed, particularly with regard to chemotherapy and radiotherapy. Last, emphasis is given to emerging strategies on how to target mutp53 for tumor therapy. Thus, this review will contribute to better understanding of the significance of mutp53 as a target for therapeutic strategies.

## Facts

- The tumor suppressor gene *TP53* is the most frequently mutated gene in human cancers.
- Approximately 80% of *TP53* mutations are missense mutations occurring within the central sequence-specific DNA binding domain, which is clustered around a few hotspot amino acid residues.
- Many mutp53 have gain-of-function properties, which are essential for tumorigenesis.
- Some small molecule compounds or peptide drugs can target tumors carrying mutp53 for treatment.

## Open questions

- Mutp53 has gain-of-function that plays a key role in promoting malignant phenotype of cancer, and what is the mechanism of its generation of gain-of-function phenotype?
- Can mutp53 be used as prognostic marker for tumors to make more accurate diagnosis, and monitor the response to treatment in cancer patients?
- There are various therapeutic strategies targeting mutp53, but what are the most effective approaches for tumor therapy?

## Introduction

*TP53* has been a hot research topic since it was first reported in 1979. To date, it is the gene with the highest correlation to human tumors identified, and the understanding of *TP53* has changed from oncogene to tumor suppressor gene [1]. *TP53* has been referred to as the “guardian of the genome” due to its role in responding to various external or internal stresses, such as DNA damage, activation of oncogenes, nutrient deprivation, and hypoxia [2–4]. Unfortunately, inactivation of *TP53* is a common event in tumorigenesis, with mutations occurring in more than 50% of human primary tumors [5]. The majority of mutations in *TP53* are missense mutations. In addition to loss of tumor suppressive function, these mutants often have gain-of-function activity and contribute to the malignant properties of cancer cells [6]. For instance, Dittmer et al. introduced p53 V143A, R175H, R248W, R273H, and D281G mutants into p53-deficient fibroblasts, resulting in enhanced tumorigenic potential in nude mice [7]. Li et al. constructed p53 K117R mutant knock-in mice, which completely abolished p53-mediated apoptosis [8]. In comparison to p53-deficient or p53 wild-type tumors, tumors carrying mutp53 exhibit more aggressive and metastatic properties [9–11]. Germline *TP53* mutations are the cause of Li-Fraumeni syndrome, which predisposes to a variety of early-onset cancers including breast carcinomas, sarcomas, brain tumors, and adrenal cortical carcinomas [12–14]. Somatic *TP53* mutations contribute to sporadic tumors such as ovarian cancer, breast cancer, colorectal cancer, head and neck cancer, and lung cancer [9, 10, 15]. More importantly, mutations in *TP53* are correlated with poor prognosis in malignancies of breast, bladder, and haematopoietic system [16–18]. Furthermore, *TP53* mutational spectrum differs among tumors [19, 20]. Herein, in this review, we summarize our understanding of mutp53 types and mutp53 spectrum in cancers. The mechanisms of mutp53 accumulation and gain-of-function are also summarized. Furthermore, we discuss the gain-of-function of mutp53 in cancers: genetic instability, ferroptosis, microenvironment, and stemness. Importantly, the role of mutp53 in the clinic is also discussed, particularly with regard to chemotherapy and radiotherapy. Last, we outline the emerging strategies to target mutp53 for tumor therapy. Therefore, this review will contribute to better understanding of the significance of mutp53 as a target for therapeutic strategies.

### Mutp53 types in cancer

**Fig. 1:**
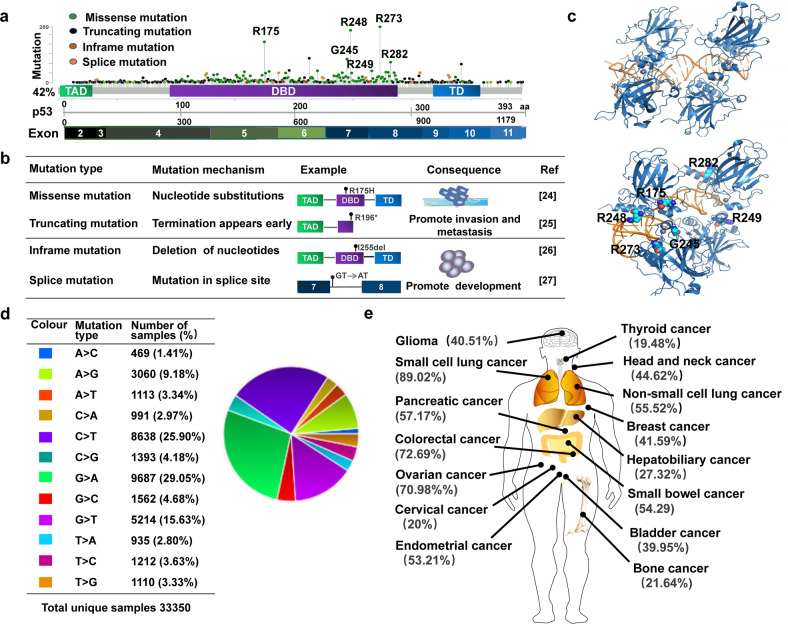
General characteristics of mutp53. **a** p53 is composed of amino-terminal transcription activation domain (TAD), DNA binding domain (DBD) and carboxy-terminal tetramerization domain (TD). The main mutant types of *TP53* include missense mutations, truncating mutations, inframe mutations, and splice mutations. **b** Different mutation types caused by different mechanisms and its impact on tumor development. **c** Structure of p53 core domain and common mutation sites (R175, G245, R248, R249, R273, R282). Figure adapted from RCSB PDB (PDB 2AC0). **d** Common substitution mutations shown in the COSMIC Database. **e** Mutation frequency of p53 in different tumors.

*TP53* is located on the short arm of human chromosome 17 (17p13.1) and consists of 11 exons, 10 introns and 393 amino acid residues. p53 protein is a transcription factor that is usually divided into three functional domains: the amino-terminal domain, the DNA binding domain and the carboxy-terminal domain [21]. Wild-type p53 (wtp53) plays pivotal role in many important biological processes by regulating the transcription of several target genes [22]. However, mutp53 not only loses the tumor suppressor function of wtp53, but also acquires new functions that contribute to the progression of malignant tumors [23]. The main mutant types of *TP53* include missense mutations, truncating mutations, inframe mutations, and splice mutations (Fig. 1a). Missense mutations result in single amino acid substitutions, which can display gain-of-function activity during tumorigenesis, such as p53 R175H and R273H mutants that promote tumor cell invasion and migration [9, 24]. Truncating mutations result in truncated proteins, which can also promote tumor development. For example, the p53 exon 6 truncating mutants R196* and R213* promote proliferation and metastasis of tumor cells [25]. Inframe mutations are caused by deletions or insertions of nucleotides [26]. Splice mutations are caused by mutations occurring at the splice site [27]. Thus, different *TP53* mutation types are caused by distinct mechanisms and contribute to the malignant development of the tumor (Fig. 1b).

Approximately 80% of *TP53* mutations are missense mutations [28]. It is mainly located in exons 5–8 (Fig. 1a), which encode the DNA binding domain, with the most common mutation sites occurring at R175, G245, R248, R249, R273 and R282 (Fig. 1c). Using the COSMIC Database (https://cancer.sanger.ac.uk/signatures/) showed that the most substitution mutations are G to A transitions, followed by C to T transitions (Fig. 1d). Missense mutations are usually divided into two categories. One category is DNA contact mutations, which occur in amino acids in contact with DNA, resulting in the inability of p53 to bind to DNA, such as p53 R273H and R248Q mutants. The other category is conformational mutations, which occur in amino acids that maintain structure, resulting in unfolded proteins, such as p53 R175H, Y220C and R249S mutants [29]. Interestingly, not all mutations are equivalent. For example, contact mutants have a lower affinity for p63 or p73 than conformational mutants [30, 31]. Mutations in the amino-terminal transactivation domain lead to truncated form of p53, which can activate apoptotic target genes [32]. However, most mutations occur in wtp53 DNA binding domain and lead to functional inactivation. Different single amino acid substitutions of the same residue also have different effects. p53 R175C mutant induces both cell cycle arrest and apoptosis, whereas p53 R175P mutant induces only cell cycle arrest and p53 R175D mutant loses both functions [3, 33]. In addition, *TP53* mutation will increase structural instability and expose adhesion sequence wrapped in the hydrophobic core of p53 to protein surface, which will drive the formation of p53 aggregates [34]. Aggregates of mutp53 are detected in high-grade serous ovarian, colorectal, and prostate cancers, resulting in loss of tumor suppressive function of wtp53 or having gain-of-function to promote tumor development [34–36]. More importantly, mutp53 can co-aggregate with p63 and p73, preventing p63 and p73 from entering the nucleus to perform transcriptional regulatory functions [37].

### Mutp53 spectrum in cancer

**Fig. 2:**
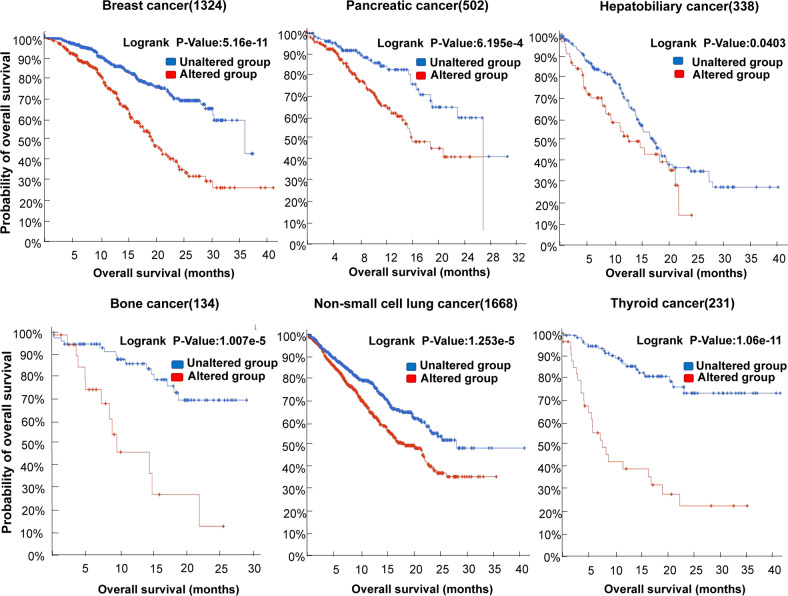
Relationship between expression of mutp53 and overall survival in cancer patients. The relationship between mutp53 expression and overall survival in cancer patients. In some tumors,mutp53 expression is associated with poorer prognosis.

Evidence suggests that the *TP53* mutational spectrum differs between tumors [38, 39]. The cBioportal for Cancer Genomics Database (https://www.cbioportal.org/) showed that frequency of *TP53* mutations in tumor tissue samples from 10,000 cancer patients is 42%. However, the mutation frequency varies across different types of tumors, with mutation frequency of 89.02% in small cell lung cancer and 72.69% in colorectal cancer. In contrast, the frequency of *TP53* mutations is lower in malignancies such as thyroid cancer, cervical cancer and bone cancer (Fig. 1e). In lung and liver cancers, G:C to T:A transversions are the most common substitutions. In colorectal cancer, brain tumors, and leukemia, transition mutations mostly occur in CpG dinucleotide hotspots. In esophageal cancer, A:T base pair mutations are more common [39]. Furthermore, mutation spectrum of *TP53* also varies among tumor subtypes in the same organ [9]. For example, Dumay et al. studied the mutational spectrum of *TP53* in 572 breast cancers and found that luminal breast cancers were predominantly missense mutations, particularly A:T to G:C transitions, whereas basal breast cancers showed a higher incidence of truncating mutations [40]. Moreover, the mutational spectrum of *TP53* in tumors is correlated with environmental carcinogens. For instance, ultraviolet light induces CC-TT double base transition in invasive squamous cell carcinomas of the skin [41]. More G to T transitions occur in smokers compared to non-smokers in lung cancer [42]. Aflatoxin B1 induces typical G:C to T:A transversions in codon 249 of p53 in primary hepatocellular carcinoma [39]. Remarkably, mutations in *TP53* are associated with poor prognosis in malignant tumors [18]. The cBioportal for Cancer Genomics Database showed that expression of mutp53 is negatively correlated with overall survival of patients in breast cancer, pancreatic cancer, hepatobiliary cancer, bone cancer, non-small cell lung cancer, and thyroid cancer (Fig. 2).

## Regulatory mechanisms of mutp53

### Mutp53 accumulation in cancer

Mutp53 is highly expressed in tumor cells, which is essential for its gain-of-function activity [43]. However, the exact mechanism of mutp53 accumulation in tumors is not fully understood. Post-translational modifications are central to many cellular signaling events and also play an essential role in regulation of p53 [44]. Wtp53 acts as a DNA sequence-specific transcriptional regulator that activates upon sensing various stress signals, and post-translational modifications can regulate its activation [45]. Similar to wtp53, post-transcriptional modifications such as phosphorylation, acetylation and ubiquitination can also regulate the level of mutp53. Studies have reported that mutp53 can be modified by phosphorylation at Ser15, Thr81 and Ser392 sites [46]. Interestingly, phosphorylation of mutp53 on Ser15/Ser37 by DNA-PK favors stabilization of mutp53 and enhances its gain-of-function activity in ovarian cancer [47]. In contrast, in prostate cancer, inhibition of NF-κB leads to phosphorylation of mutp53 at Ser15, thereby restoring DNA binding capacity [48]. Moreover, mutp53 can be modified by acetylation. Overexpression of TRRAP, a constituent of several histone acetyltransferase complexes, increases mutp53 levels, whereas silencing TRRAP reduces mutp53 accumulation in lymphoma and colon cancer [43]. Besides phosphorylation and acetylation, ubiquitination is also implicated in the regulation of mutp53. Under normal conditions, wtp53 is kept at low levels under negative feedback regulation by MDM2, which targets p53 for proteasome-mediated degradation. But mutp53 does not effectively activate MDM2, resulting in the loss of the negative regulatory role of MDM2 [49]. However, Terzian et al. found that loss of MDM2 stabilizes the p53 R172H mutant [50]. Other E3 ubiquitin ligases such as CHIP, COP1 and Pirh2 can ubiquitinate and degrade mutp53 [51, 52]. The accumulation of mutp53 in human tumors is also associated with co-chaperon and chaperon proteins such as BAG5, Hsp90, and Hsp70. BAG5 protects mutp53 from ubiquitinated degradation by MDM2 and CHIP [53]. Hsp90 and Hsp70 through interaction with the DNA binding domain of mutp53, thereby maintaining stability of mutp53 in cancer [54].

### Mutp53 exerts gain-of-function

**Fig. 3:**
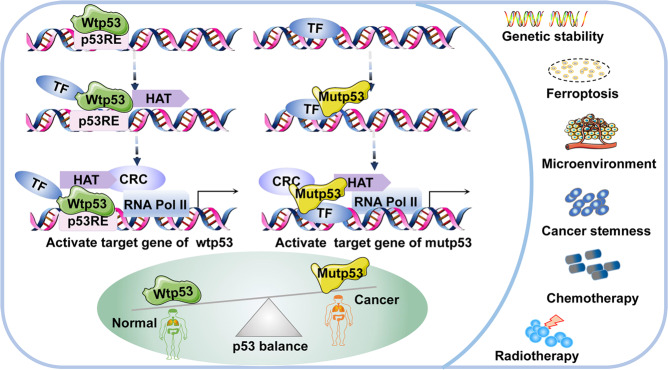
The transcriptional model of mutp53 and its function in tumors. Transcriptional model of mutp53 and its function in tumors. In contrast to wtp53, mutp53 cannot bind directlyto DNA RE and it exerts function through interactions with TFs.

Various p53 mutants utilize distinct mechanisms to exert gain-of function. To begin with, mutp53 binds to transcription factors (TFs) in order to perform its function (Fig. 3). Wtp53 recognizes and binds to DNA response elements (RE), then recruits TFs, histone acetyltransferases (HATs) such as p300, chromatin remodeling complexes (CRCs) such as SWI and SNF that bind to acetylated histones [21, 55], and RNA polymerase II, which binds to open promoters to form the pre-initiation complex (Fig. 3). However, it was reported that mutp53 cannot bind to the p53 DNA RE, and it exerts its gain-of-function activity through different mechanisms to promote tumorigenesis. For instance, mutp53 binds to diverse TFs and cofactors such as NF-Y, p73, NRF2, Ets-1, and regulates the transcription of their target genes [55]. In response to DNA damage, mutp53 binds to NF-Y target promoters and recruits p300 to acetylate histones, resulting in overexpression of cell cycle genes and promoting malignant tumor development [56]. In some circumstances, mutp53 can bind to some specific structures of DNA and regulate transcription, such as matrix attachment regions [57]. Also, mutp53 can interact with other proteins, thereby altering or inhibiting their function. In colorectal and pancreatic cancers, mutp53 antagonizes p63/p73-mediated tumor suppression via the Notch1 signaling pathway [58]. Notably, appropriate cellular localization of mutp53 also contributes to its gain-of-function. Mutp53 is usually located in the nucleus, but in some cases it is located in the cytoplasm, which may be related to the types of mutation [16]. For example, Morselli et al. found that p53 P151H and R282W mutants were located in the nucleus and could not inhibit autophagy, whereas p53 E258K, R273H and R273L mutants were located in the cytoplasm and could inhibit autophagy in colon cancer [59].

### Mutp53 transgenic mouse models

Mutations in *TP53* found in human cancer are compiled in the IARC *TP53* Database (http://www-p53.iarc.fr/), which provides cell and mouse models for cancer research [13]. The p53 transgenic mouse model has been widely used to explore the biological functions of p53 and contribute to understand the role of p53 in tumorigenesis in vivo [60]. p53 knockout mice demonstrate that p53 is critical for preventing tumorigenesis. For instance, p53 knockout mice are sensitive to carcinogens such as dimethylnitrosamine induced tumors [61]. Furthermore, in the context of 129/sv and C57BL/C, p53-/- mice develop tumors earlier than p53+/- mice. T-cell lymphomas frequently occur in p53-/- mice, whereas osteosarcoma and soft tissue sarcoma mostly occur in p53+/- mice [][60]. In mouse models of pancreatic and lung cancer, loss of p53 regulates the tumor microenvironment, which promotes the accumulation of suppressor Treg cells as well as impairs Th1 and CD8 + T cell responses [62].

However, mutp53 knock-in mouse model further demonstrates the gain-of-function of mutp53. Duan et al. constructed an SP-C/p53 R273H transgenic mouse model for studying the role of mutp53 in lung tumorigenesis. SP-C/p53 R273H transgenic mice had an increased incidence of adenocarcinoma and accelerated age of onset compared to age-matched non-transgenic littermates [63]. Compared to p53-/- cells, p53 R175H mutant knock-in mice result in chromosomal translocations and G2/M checkpoint defects. More importantly, the tumor spectrum observed in p53 R175H mutant mice is more complex than in p53-/- mice. Thymic tumors and sarcomas are commonly observed in both p53 R175H and p53-/- mice, but peripheral lymphomas and germ-cell tumors are only observed in p53 R175H mice [64]. Interestingly, hot spot mutp53 mouse models display differential gain-of-function in tumorigenesis. Compared to p53-null mice, p53 R248Q/- mice have stronger gain-of-function, which accelerates tumor onset and shorter survival. In contrast, p53 G245S/- mice are similar to null mice in terms of tumor latency and survival in the 129 Sv/C57BL6 background [11]. Moreover, compared to p53+/- mice, p53 R270H/+ mice have an increased incidence of carcinomas and B-cell lymphomas. p53 R172H/+ mice are more susceptible to metastatic osteosarcoma in the 129S4/SvJae background [65]. Though p53+/515 A mice displays similar tumor spectrum and survival curves to p53+/- mice, p53+/515 A mice show a high frequency of tumor metastasis in the C57BL6 background [66]. The p53 knock-in and knockout mouse model models mimic initiating events in human tumorigenesis and progression, which are essential for preclinical studies [60].

## Gain-of-function of mutp53 in cancer

### Genetic instability

Genomic instability is suggested to be a feature of human cancers [67]. Wtp53 plays an important role in maintaining genome stability as the guardian of genome, whereas mutp53 can promote genome instability (Table 1). Mutp53 was found to promote amplification and chromosomal instability [67–69]. For instance, mutp53 promotes gene amplification by interacting with topoisomerase I in osteosarcoma [67]. In pre-tumor thymocytes, mutp53 induces inter-chromosomal translocation [69]. In lung cancer, mutp53 facilitates formation of DNA replication origins and stabilizes replication forks, which leads to formation of micronuclei and proliferation of genomically abnormal cells [70]. Also, mutp53 inhibits binding of the MRE11–RAD50–NSB1 complex to sites of DNA damage, resulting in ATM inactivation and genetic instability [69]. In breast cancer and lung cancer, mutp53 suppresses expression of BRCA1 and RAD17, which prevents DNA damage repair and causes genomic instability [71]. Notably, cell-in-cell structures have been identified in many solid tumors, wtp53 promotes death of cells that form these structures, whereas mutp53 contributes to formation of cell-in-cell structures in lung adenocarcinoma through live cell engulfment, leading to abnormal mitosis [72]. Thus, the crosstalk between mutp53 and genome instability is critical to cancer development.

**Table 1:** The function of mutp53 and the corresponding regulatory mechanisms.

Role	Tumor type	Target	Mechanism	Ref
Genetic instability	Osteosarcoma	Topoisomerase I	Promotes gene amplification	[67] [69]
		Mre11	Causes ineffective activation of ATM and genetic instability	
	Lung cancer	Cyclin A, CHK1	Promotes proliferation of cells with genomic abnormalities	[70]
	Breast cancer	E2F4	Inhibits DNA damage repair	[71]
	Lung adenocarcinoma	Cell in cell	Causes abnormal mitosis and genomic instability	[72]
Ferroptosis	Lung cancer	SLC7A11	p53 ^3KR^ induces cell ferroptosis	[76]
			p53^4KR98^]does not induce ferroptosis	[82]
	Oesophageal cancer	NRF2	Causes cell ferroptosis	[79]
Microenvironment	Colon cancer	TAM	Renders macrophages in a pro-tumor state	[92]
	NSCLS	ID4	Leads to tumor angiogenesis	[93]
	Leukemia	VEGF	Leads to tumor growth and metastasis	[94]
	Breast cancer	NF-κB	Causes an increased inflammatory response	[95]
	Colon adenocarcinoma	sIL-1Ra	Generates a pro-inflammatory tumor microenvironment	[96]
Stemness	Colon cancer	Lgr5, CD44	Promotes the expression of markers of CSCs	[99]
	Glioblastoma	WIP	Increases the expression of CSC-like markers	[100]
	Colorectal cancer	lncRNA	Enhances the stemness	[101]
	Breast cancer	MiR-200c	Promotes cancer stemness	[102]
	Lung adenocarcinoma	MiR-324-5p	Promotes cancer stemness	[103]
Clinical	Colorectal cancer	Bax, Bak, VDAC	Inhibits apoptosis of tumor cells	[105]
		EFNB2	Causes chemoresistance	[111]
	Osteosarcoma	Procaspase-3	Causes chemoresistance	[108]
	Colon cancer	PUMA	Causes chemoresistance	[109]
		p73	Inhibits apoptosis of tumor cells	[110]
		MDR1	Causes chemoresistance	[112]

### Ferroptosis

**Fig. 4:**
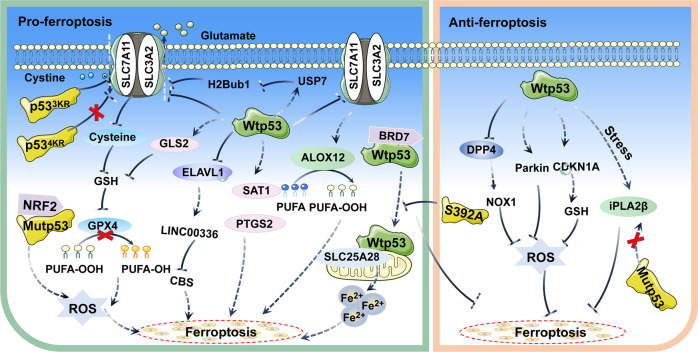
Schematic representation of the mechanism of mutp53 in ferroptosis. p53 can regulate the ferroptosis pathway through diverse mechanisms. In most cases, p53 promotes ferroptosis. However, in certain circumstances, p53 can inhibit the onset of ferroptosis.

Ferroptosis is an iron-dependent form of cell death that has been reported to inhibit tumor growth as an independent pathway [73–75]. Interestingly, p53 was found to have a critical but complex role for the regulation of ferroptosis. Although most studies have supported the function of p53 in promoting ferroptosis. In certain circumstances, p53 can inhibit ferroptosis (Table 1) (Fig. 4). In lung cancer, wtp53 inhibits cystine uptake by suppressing expression of SLC7A11, leading to reduced activity of GPX4 and cellular antioxidant capacity, which causes the onset of ferroptosis [76]. Wtp53 also inhibits the level of H2Bub1 by promoting nuclear translocation of the deubiquitinase USP7, further contributing to the inactivation of SLC7A11 expression [77]. Furthermore, wtp53 induces ALOX12 expression by downregulating SLC7A11 levels, resulting in ALOX12-dependent ferroptosis [78]. In esophageal and lung cancers, mutp53 suppresses SLC7A11 expression by interacting with the master antioxidant transcription factor NRF2, which promotes the accumulation of ROS and induces ferroptosis [79]. Notably, Jiang et al. replaced lysine residues at sites 117,161 and 162 of p53 with arginine residues in tumor cells to construct acetylation-deficient p53 ^3KR^ mutant mice, which did not regulate cell cycle and apoptosis like wtp53, but inhibited SLC7A11 expression and induced ferroptosis [76, 80]. In tumors carrying mutp53, ectopic expression of SLC7A11 promotes tumor resistance to drugs that induce ferroptosis, further suggesting that mutp53 sensitizes cancer cells to ferroptosis by inhibiting SLC7A11 [79]. In the DNA double stand break repair genes XRCC4 knockout background, p53 ^3KR^ mice exhibit senescence-like phenotypes, and p53-mediated ferroptosis is greatly induced in the testis of this mouse, so the combination of ferroptosis and genomic instability may significantly promote senescence [81]. However, Wang et al. constructed the p53 ^4KR^ mutant mice (K98R + 3KR), which were not only defective in inhibiting tumor growth, but also failed to inhibit expression of SLC7A11 and induce ferroptosis. Compared with p53 ^3KR^ mice, p53 ^4KR^ mice can develop tumors earlier [82]. Additionally, in hepatic stellate cells, wtp53 is translocated to mitochondria through binding to BRD7 and interacts with SLC25A28, which leads to abnormal accumulation of redox-active iron and promotes ferroptosis. In contrast, p53 S392A mutant blocks the binding of BRD7 to p53, which in turn prevents the mitochondrial translocation of p53 and inhibits the onset of ferroptosis [83]. In lung cancer, wtp53 regulates the level of LncRNA LINC00336 by suppressing ELAVL1 expression, which decreases the expression level of cystathionine-β-synthase (CBS) and promotes ferroptosis [84]. Wtp53 also induces ferroptosis by regulating the expression of SAT1, GLS2, and PTGS2 [85, 86]. Interestingly, wtp53 can inhibit the onset of ferroptosis. For instance, wtp53 can inhibit ferroptosis by activating the expression of iPLA2β at low levels of stress in lung cancer, but the activation of wtp53 is diminished at high levels of stress. In contrast, p53 R175H, R273H and R248W mutants do not readily induce the expression of iPLA2β [87]. In colorectal cancer, wtp53 inhibits ferroptosis by blocking DPP4 activity in a transcription-independent manner [88]. In fibrosarcoma, wtp53 can regulate the expression of CDKN1A to delay the onset of ferroptosis in response to cystine deprivation [89]. Wtp53 also may limit cystine deprivation-induced ferroptosis by activating Parkin expression and reducing ROS levels [90]. Thus, these findings suggest that p53 can regulate ferroptosis, which has significant implications for the treatment of tumors.

### Tumor microenvironment

Increasing evidence suggests that mutp53 can regulate the tumor microenvironment (Table 1). Tumor-associated macrophages (TAM) are the hallmark of solid tumors. Wtp53 suppresses tumorigenesis by promoting an anti-tumor microenvironment and modulates M1 polarization pattern in neighboring macrophages [91]. Interestingly, in colon cancer, mutp53 selectively releases miR-1246-rich exosomes that are taken up by surrounding macrophages, leading to miR-1246-dependent reprogramming into a tumor-promoting M2 state [92]. Mutp53 can also promote tumor neo-angiogenesis. In non-small cell lung cancer (NSCLS), ID4 protein promotes expression of pro-angiogenic factors IL8 and GRO-α. However, mutp53 activates ID4 and depletion of mutp53 impairs ID4 expression [93]. In leukemia, mutp53 can promote synthesis of VEGF, providing favorable environment for cell growth [94]. Moreover, chronic inflammation is also a characteristic of tumors. In breast cancer, mutp53 affects TNF-induced activation of NF-κB, which exacerbates the inflammatory response [95]. In colon adenocarcinoma, mutp53 can produce pro-inflammatory tumor microenvironment by suppressing expression of sIL-1Ra, leading to tumor malignancy [96].

### Cancer stemness

Mutp53 was found to contribute to the acquisition of cancer stem cells (CSCs) phenotype (Table 1). The hallmark feature of CSCs is their ability to produce heterogeneous tumor cells, which are critical in the initiation and progression of cancer [97]. Wtp53 usually serves as a barrier to CSCs formation and inhibits the expression of CSCs markers [98]. However, mutp53 promotes the expression of CSCs markers such as CD44, Lgr5, and ALDH, and enhances the expansion of CSCs sub-populations to promote colorectal cancer development [99]. In glioblastoma and breast cancer, overexpression of mutp53 not only increases the expression of CSCs markers, but also promotes the proliferation of CSCs [100]. Additionally, p53 R273H mutant can regulate the expression of lncRNAs such as lnc273-31 and lnc273-34 in colorectal cancer, promoting CSCs self-renewal and tumor proliferation [101]. Mutp53 also promotes cancer stemness by regulating miRNA. For instance, mutp53 promotes cancer stemness through modulating miR-200c-PCK2 axis in basal-like breast cancer [102]. In lung adenocarcinoma, mutp53 facilitates cancer stemness via regulating miR-324-5p-CUEDC2-NF-κB pathway [103]. These findings suggest that mutp53 can regulate cancer stemness, thereby providing new direction for treatment of tumors.

## Clinical impact of mutp53 in cancer

### Chemotherapy

**Fig. 5:**
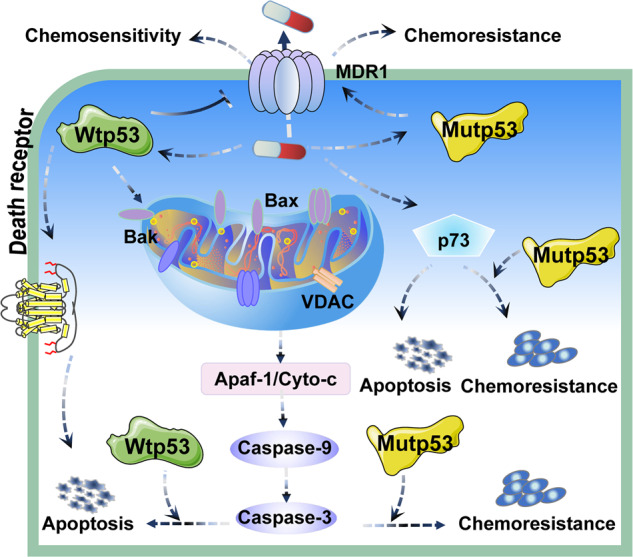
Schematic representation of the mechanism of mutp53 in chemotherapy. Expression of mutp53 is positively correlated with increased resistance to chemotherapy in different tumors.

Chemotherapy is an integral part of cancer treatment, but chemoresistance has become a major barrier to treatment. Plenty of evidence suggests that expression of mutp53 is positively correlated with increased chemoresistance in different tumors (Table 1) (Fig. 5). Induction of apoptosis is one of the most important functions of p53, and disruption of this function can promote tumor chemoresistance [104]. Wtp53 can induce apoptosis through mitochondrial and Fas-mediated apoptotic pathways [105, 106]. As shown in Fig. 5, wtp53 induces oligomerization of Bax, Bak and VDAC, increases the permeability of the outer mitochondrial membrane and promotes the release of cytochrome c. Chemotherapeutic agents such as 5-fluorouracil and oxaliplatin sensitize colorectal cancer cells carrying wtp53 to Fas-mediated apoptosis [107]. In contrast, p53 R175H, L194F, R249S, and R280K mutants lose the ability to activate the Bax/Bak lipid pore and alter VDAC multimerization state, which inhabit apoptosis in cancer cells [105]. In osteosarcoma, p53 R273H mutant reduces expression of procaspase-3, resulting in failure of chemotherapeutic agents such as methotrexate and doxorubicin to induce apoptosis [108]. In colon cancer, mutp53 does not bind to PUMA promoter to activate its transcription, which helps tumor cells evade apoptosis and reduces sensitivity to 5-fluorouracil [109]. Furthermore, in tumor cells lacking functional p53, various chemotherapeutic agents can cause apoptosis by inducing expression of p73. Yet, mutp53 can inactivate p73 in colon cancer, and downregulation of mutp53 enhances chemosensitivity [110]. In colorectal cancer, mutp53 activates EFNB2 in response to DNA damage, while silencing EFNB2 increases the sensitivity of cancer cells to 5-fluorouracil [111]. Additionally, studies have found that high expression of MDR1 in different tumors is significantly correlated with chemoresistance. For instance, in colon cancer and osteosarcoma, mutp53 specifically upregulates MDR1 expression by interacting with Ets-1, which leads to chemoresistance [112]. In colorectal cancer, 5-fluorouracil promotes the expression of p53. However, in contrast to wtp53, mutp53 fails to inhibit LRPPRC expression after DNA damage, resulting in an increase in MDR1 transcription, which leads to chemoresistance [113]. Thus, these findings suggest that mutp53 plays a crucial role in regulating chemoresistance of tumor cells.

### Radiotherapy

**Fig. 6:**
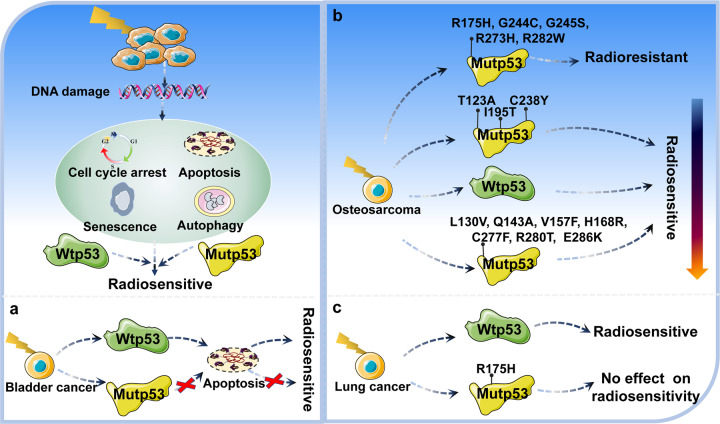
Schematic representation of the mechanism of mutp53 in radiotherapy. Mutp53 can regulate radiotherapy through various mechanisms. In most cases, expression of mutp53 leads to radiotherapy resistance. However, under a certain context, mutp53 expression can promote radiotherapy sensitivity or have no effect on radiotherapy sensitivity.

Radiotherapy is now considered to be one of the effective approaches to cancer treatment. However, many tumors exhibit resistance to radiation [114]. Hence, it is critical to determine the role of p53 status in radiotherapy (Fig. 6). In diffuse intrinsic pontine gliomas, mutations in p53 are a major driver of increased radiation resistance, with mutp53 carrying patients less responsive to irradiation and relapsing earlier after radiotherapy with a worse prognosis [114]. O’Connor et al. studied the response of p53 status to radiation in 60 different cancer cell lines. In contrast to cell carrying wtp53, most tumor cells carrying mutp53 failed to induce expression of CIP1/WAF1, GADD45 and MDM2 mRNA, as well as G1 phase arrest after γ-irradiation, resulting in radioresistance [115]. In bladder cancer, ionizing radiation can induce tumor cells carrying wtp53 to undergo G1 phase arrest and apoptosis, resulting in a higher radiosensitivity. In contrast, it is not significantly observed in tumor cells carrying mutp53 (Fig. 6a) [116]. Kuerbitz et al. further demonstrated that mutp53 lost the ability to induce G1 phase arrest after γ-irradiation [117]. In glioblastoma, clonogenic survival assays have shown that U87 cells carrying wtp53 and T98 cells carrying mutp53 exhibit essentially identical sensitivity to fractionated radiotherapy. But cells carrying wtp53 in response to ionizing radiation exhibit accelerated senescence [118]. In ovarian cancer, cells carrying wtp53 are very sensitive to irradiation, which leads to p53 accumulation after irradiation, whereas cells carrying mutp53 show varying degrees of radiation resistance and do not lead to p53 accumulation after irradiation [119]. In head and neck cancer [120], hepatocellular carcinoma [121], cervical cancer [122], and endometrial cancer [123], cells carrying mutp53 are also more resistant to radiation. Furthermore, transgenic mice carrying mutp53 increases resistance of various hematopoietic cell lineages to γ-irradiation, and overexpression of p53 R193P or A135V mutants increases radiation resistance of mouse hematopoietic cell by 45–57% [124].

Notably, the relationship between mutp53 and radiosensitivity is controversial, since certain studies have shown that mutp53 can increase radiosensitivity or have no effect on radiosensitivity [125]. For instance, Kawashima et al. introduced the p53 R273H mutant into immortalized human fibroblasts and found that cells carrying the p53 R273H mutant had higher radiosensitivity than cells not expressing p53 after X-ray irradiation [125]. In rat lung embryonic epithelial cells, compared to cells carrying wtp53, cells carrying mutp53 display significantly lower survival after γ-irradiation at doses of 2 to 12 Gy, suggesting that mutations in the p53 increase sensitivity to ionizing radiation [126]. Interestingly, different mutant sites of p53 are differentially sensitive to radiotherapy [127]. In osteosarcoma, after γ-irradiation treatment of cell lines, p53 mutations at codons 175, 244, 245, 273, and 282 are radioresistant. Mutations at codons 123, 195, and 238 have higher radiosensitivity than wtp53, and mutations at codons 130, 143, 157, 168, 277, 280, and 286 are less radiosensitive than wtp53 (Fig. 6b) [127]. Phosphorylation modifications also affect sensitivity to radiotherapy. In lung cancer, cells carrying p53 S15A and S46A mutants are radiosensitive, whereas cells carrying p53 S15D, S20A and S20D mutants are medium radiosensitive [128]. Furthermore, Tada et al. determined the status of p53 by sensitive yeast functional assay in a study of 36 patients with glioblastoma treated with radiotherapy, and found that patients carrying mutp53 had a significantly longer regrowth-free period after treatment [129]. However, wtp53 effectively abrogates ionizing radiation-induced autophagy and activates apoptosis to regulate radiosensitivity in lung cancer, while p53 R175H mutant has no effect on radiosensitivity (Fig. 6c) [130]. Thus, further research is needed to determine the link between mutp53 and radiotherapy, which is of great significance for treatment of patients.

## Strategies of tumor treatment

### Restoration of wild-type activity

**Fig. 7:**
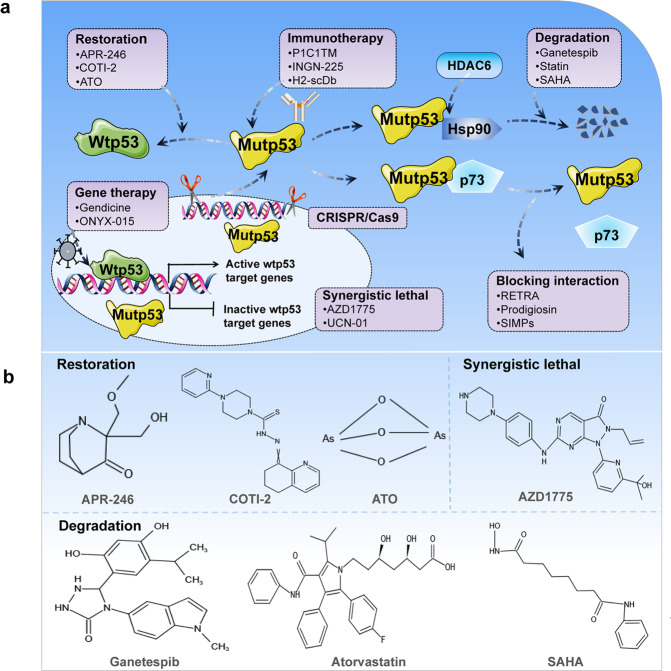
Schematic representation of the mechanism of targeting mutp53 for tumor therapy. **a** Treatment strategies for tumor cells carrying mutp53. **b** Chemical structures of common drugs used in clinical trials.

Reactivation of wild-type activity of mutp53 is an effective strategy to slow tumor progression (Fig. 7a). Many studies have found that small molecule compounds and peptide drugs can induce changes in the spatial conformation and folding pattern of mutp53, such as CP-31398 [131], RITA [132], PEITC [133], NSC319726 [134], Chetomin [135], ReACp53 [34, 136], and pCAPs [136]. Of note, APR-246, COT1-2, PC14586, and Arsenic Trioxide (ATO) are currently undergoing clinical trials (Table 2) (Fig. 7b). APR-246 is also known as PRIMA-1^MET^, and its active form in vivo is methylene quinuclidinone. It restores wild-type conformation and anti-tumor transcriptional activity by covalently binding the DNA binding domain of mutp53 [137, 138]. APR-246 has significant anti-tumor activity in esophageal adenocarcinoma, acute myeloid leukemia, and triple-negative breast cancer [139–141]. Combining APR-246 with multiple anti-cancer drugs can enhance the effectiveness of treatment. Liu et al. found that APR-246 combined with cisplatin and 5-fluorouracil can enhance the inhibitory effect on esophageal adenocarcinoma [139]. Furthermore, studies have found that APR-246 displays mutp53 non-dependent effects, which induce elevated ROS through depletion of glutathione content, ultimately triggering lipid peroxidation cell death [79]. Additionally, COTI-2 can reactivate mutp53 and restore DNA binding properties, which inhibit cell growth and induce apoptosis [142]. PC14586 is a reactivator of p53 Y220C mutation, which is currently in clinical trials [143]. ATO can target structural mutp53 and restore transcriptional activity. Mouse xenograft models also demonstrate that ATO reactivates mutp53 to suppress tumors [144]. Thus, these studies suggest that restoring mutp53 to wild-type conformation is a promising anti-cancer strategy.

**Table 2:** Clinical trial drug for targeting cancer cells carrying mutp53.

Role	Drug	Disease	Phase	NCT number	Start date
Restoration	APR-246	High-grade serous ovarian cancer	Phase Ib/II	NCT02098343	March 2014
			Phase II	NCT03268382	July 2017
		Oesophageal carcinoma	Phase Ib/II	NCT02999893	April 2017
		AML or MDS	Phase II	NCT03931291	September 2019
		MDS	Phase III	NCT03745716	January 2019
		Myeloid malignancy	Phase I	NCT04214860	December 2019
		Myeloid neoplasms	Phase Ib/ II	NCT03588078	September 2018
	COTI-2	Advanced or recurrent malignancies	Phase I	NCT02433626	December 2015
	PC14586	Advanced solid tumor, advanced malignant neoplasm, metastatic cancer, metastatic solid tumor	Phase I/ II	NCT04585750	October 2020
	ATO	AML or MDS	Phase I	NCT03855371	January 2018
		Refractory cancer, intractable cancer	Phase II	NCT04695223	January 2021
		Refractory solid tumors	Phase II	NCT04869475	April 2021
Degradation	Ganetespib	Epithelial ovarian cancer, fallopian tube cancer, primary peritoneal cancer	Phase I/ II	NCT02012192	July 2014
	Atorvastatin	Solid tumor and relapsed AML	Phase I	NCT03560882	July 2018
		Colorectal carcinoma, ulcerative colitis	Phase II	NCT04767984	September 2021
	SAHA	Advanced cancers	Phase I	NCT02042989	June 2014
Synergistic	AZD1775	Small cell lung cancer	Phase II	NCT02688907	June 2016
		Advanced gastric adenocarcinoma	Phase II	NCT02448329	January 2015
		Ovarian cancer	Phase II	NCT01357161	July 2011
		Epithelial ovarian cancer	Phase II	NCT01164995	July 2010

### Degradation of mutp53

Mutp53 can form stable aggregates that accumulate in cells and play an important role in cancer progression [145]. Therefore, promoting the degradation of mutp53 may also exhibit antitumor effects (Fig. 7a). Some drugs such as gambogic acid [146], capsaicin [147], MCB-613 [145], and NSC59984 can degrade mutp53 [148]. Of note, ganetespib, statin, and SAHA are in clinical trials (Table 2) (Fig. 7b). The Hsp90/HDAC6 chaperone mechanism is a major determinant in stabilizing mutp53. Ganetespib is >50-fold more potent than the first generation Hsp90 inhibitor 17AAG in degrading and killing cancer cells carrying mutp53 [149]. In various hematological and solid tumors, ganetespib exhibits potent cytotoxicity [150]. Furthermore, treatment of p53 R172H/R172H and p53 R248Q/- mice with ganetespib inhibits tumor growth and prolongs survival in a mutp53-dependent manner, but it has no effect on p53-null mice [149]. Ganetespib can be used in combination with chemotherapy agent cyclophosphamide to have a better inhibitory effect on tumor growth [151]. Additionally, statins are degradation inducers of conformational or misfolded mutp53, which induce CHIP-mediated mutp53 degradation by inhibiting the interaction of mutp53 with DNAJA1, but have little effect on wtp53 and DNA contact mutants [51]. Moreover, SAHA shows preferential cytotoxicity in cancer cells carrying mutp53. It interferes with the interaction between Hsp90 and mutp53 by inhibiting HDAC6, which in turn causes the reactivation of MDM2 and CHIP, thus exerting ability to degrade mutp53 [152]. Thus, these studies suggest that degradation of mutp53 is another therapeutic strategy, but more clinical trials are still needed to confirm it.

### Tumor immunotherapy

Accumulating evidence suggests that p53 can regulate innate and adaptive immune responses [153, 154]. Wtp53 is an important component of Toll-like receptor 8-mediated immune response [155]. Wtp53 is also involved in the activation of the MHC-I antigen presentation pathway by inducing TAP1 [154]. However, mutations in *TP53* affect T cell recruitment and activity, leading to immune evasion and promoting cancer progression [153]. In lung cancer, mutp53 inhibits the formation of the STING-TBK1-IRF3 complex, leading to inactivation of the innate immune signaling pathway [156]. Interestingly, mutp53 has been found to have immunogenicity and can act as a neoantigen to trigger an immune response [157]. For instance, in lung adenocarcinoma, mutp53 promotes PDL-1 expression and infiltration of CD8 + T cells, as well as enhances tumor immunogenicity. Thus, patients carrying mutp53 may be more sensitive to PD-1 blockade immunotherapy [158]. In ovarian cancer and metastatic colorectal cancer, there are specific T cells against the mutant neoantigen in tumor infiltrating lymphocytes [159, 160], which can be used for adoptive cell therapy. Additionally, P1C1TM is an engineered T cell receptor-like antibody that differentiates between mutp53 and wtp53 expressing HLA-A24^+^ cells and mediates antibody dependent cellular cytotoxicity in cells carrying mutp53 (Fig. 7a). The combination of P1C1TM with PNU-159682 specifically suppresses growth of tumor [161]. H2-scDb is a bispecific antibody that can specifically recognize cancer cells carrying p53 R175H mutant, and effectively activate T cells to lyse tumor cells in vitro and in vivo [162]. In addition to antibodies, tumor vaccines also play an essential role in immunotherapy. INGN-225 is a p53-modified adenovirus-mediated dendritic cell vaccine (Fig. 7a). In a phase II clinical trial for small cell lung cancer, INGN-225 was shown to induce a significant immune response and improve efficacy of chemotherapy [163]. Thus, understanding the role of mutp53 in immune regulation will help develop more effective antitumor immunotherapies.

### Other therapies

Numerous studies have found that interfering protein interactions, synergistic lethal therapies, gene therapy and genomic editing can also be used as therapeutic strategies for targeting mutp53 (Fig. 7a). Mutp53 exerts gain-of-function by interacting with many proteins. Hence, interfering with protein interaction can also be a strategy. RETRA, a small molecule compound, can release p73 from mutp53-p73 complex, which inhibits tumor development [164]. Prodigiosin not only disrupts interaction between mutp53 and p73, but also upregulates p73 expression, thus exerting antitumor effects [165]. In addition to small molecule compounds, short peptides can also interfere with interactions between mutp53 and p73. For instance, Di Agostino et al. showed that SIMPs disrupted interaction between mutp53 and p73, restored ability of p73 to mediate transcription and apoptosis, and more importantly, potentiated sensitivity of tumor cells carrying mutp53 to adriamycin and cisplatin [166]. In addition, under normal circumstances, the cell will rely on wtp53-induced G1 phase block for repair when DNA is damaged. Interestingly, when *TP53* is mutated, cancer cells will rely on G2-M checkpoints to repair damaged DNA [57, 167]. Therefore, in human cancers with *TP53* mutations, AZD1775 and UCN-01 are commonly used as synthetic lethal agents. AZD1775 is a potent and selective WEE1 inhibitor that has entered phase II clinical trials (Table 2) (Fig. 7b). Studies have demonstrated that it improves efficacy of carboplatin in treatment of ovarian cancer carrying mutp53 [168]. UCN-01 is a selective protein kinase C inhibitor. It enhances toxicity of mitomycin in human epidermal cell carcinoma and pancreatic cancer carrying mutp53 [169], and the combination of UCN-01 and inotuzumab ozogamicin markedly increases cell death [170].

Delivery of wtp53 into cancer cells via adenovirus is a direct strategy that rescues p53 activity in cancer. Gendicine is the first gene therapy product approved for the treatment of various types of cancers including head and neck cancer, lung cancer, breast cancer, cervical cancer, ovarian cancer, liver cancer, and pancreatic cancer [171, 172]. Gendicine combined with chemotherapy and radiotherapy usually produces significantly higher response rates than standard therapy alone [171]. More importantly, the mutational status of p53 does not significantly affect the outcome and long-term survival of patients treated with Gendicine [171, 172]. ONYX-015, an adenovirus with the E1B region deleted, can replicate in wtp53-deficient cancer cells and produce cytolysis. Compared to mice carrying wtp53, treatment with ONYX-015 significantly improves the survival of mice carrying mutp53 [173]. CRISPR/cas9-mediated gene editing appears to be a direct therapeutic strategy for tumor cells carrying mutp53. Chira et al. proposed a highly tumor specific *TP53* delivery system based on CRISPR/Cas9 genome editing technology that can replace mutant *TP53* in the tumor genome with a functional copy by homologous recombination, thus restoring normal p53 phenotype in tumor cells [174]. Zhan et al. constructed a p53 genetic sensor that specifically detects wtp53 expression in cells. Combining the p53 sensor with diphtheria toxin using the CRISPR/Cas9 system can specifically kill p53-deficient tumor cells [175]. Moreover, in prostate cancer, the *TP53* 414delC mutation has been repaired to the wild-type *TP53* genotype by using the CRISPR/Cas9 system, thereby promoting apoptosis and inhibiting tumors proliferation [176].

## Conclusions

There is an extremely high probability of *TP53* mutations occurring in clinical tumors. From a large amount of experimental data, it is becoming increasingly clear that mutp53 plays a key role in promoting the malignant phenotype of cancer. Hence, it is widely regarded as an attractive target for the treatment of multiple cancers. However, there are still many outstanding issues. Firstly, *TP53* is mutated in more than 50% of tumors, so what are the factors that influence the mutation types and mutation spectrum of *TP53*? Secondly, post-translational modifications play an important role in the accumulation of mutp53. How does post-translational modification regulate mutp53 to exert gain-of-function and what are its specific regulatory mechanisms? Thirdly, the current study mainly focuses on mutational hotspots of *TP53*. It is uncertain whether mutations in *TP53* with different residues and different functional domains exert the same gain-of-function [177], as well as what is the mechanism by which it exerts gain-of-function? Last but not least, mutp53 is generally considered “undruggable”. However, in recent years, although studies have reported that a variety of small molecule compounds or peptide drugs targeting mutp53 have been developed, only a few drugs have entered clinical trials, and no drugs targeting mutp53 have been approved for clinical tumor treatment. Obviously, there is still more research to be done on mutp53 in the future.
